# Pesticidal Toxicity of Phosphine and Its Interaction with Other Pest Control Treatments

**DOI:** 10.3390/cimb45030161

**Published:** 2023-03-17

**Authors:** Saad M. Alzahrani, Paul R. Ebert

**Affiliations:** 1 Advanced Agricultural & Food Technology Institute, King Abdulaziz City for Science and Technology (KACST), P.O. Box 6086, Riyadh 11442, Saudi Arabia; 2 School of Biological Sciences, The University of Queensland, St. Lucia, QLD 4072, Australia

**Keywords:** phosphine, phosphine toxicity, synergism, phosphine-resistance, cross-resistance, ionizing radiation, UV radiation

## Abstract

Phosphine is the most widely used fumigant for stored grains due to a lack of better alternatives, all of which have serious shortcomings that restrict their use. The extensive use of phosphine has led to the development of resistance among insect pests of grain, which threatens its status as a reliable fumigant. Understanding the mode of action of phosphine as well as its resistance mechanisms provides insight that may lead to improved phosphine efficacy and pest control strategies. The mechanisms of action in phosphine vary from disrupting metabolism and oxidative stress to neurotoxicity. Phosphine resistance is genetically inherited and is mediated by the mitochondrial dihydrolipoamide dehydrogenase complex. In this regard, laboratory studies have revealed treatments that synergistically enhance phosphine toxicity that may be used to suppress resistance development and enhance efficacy. Here, we discuss the reported phosphine modes of action, mechanisms of resistance and interactions with other treatments.

## 1. Introduction

Grains account for more than half of human nutrition and also provide feed for poultry and livestock. Global population growth has increased the demand for cereal crops, posing new challenges to grain storage practices [1], especially with the ideal environment that grain in silos provide for pests such as insects, mites, and rodents [2]. Insect pests in stored commodities are a serious threat and can cause major losses to the grain industry. For example, $84 million worth of grain has been rejected due to the detection of live insects [3]. To overcome this, effective pest management practices should be developed and implemented in order to reduce or eliminate pest infestation.

Due to its effectiveness and economic viability, chemical control is the preferred method for pest control in stored grain [4]. Fumigants are currently preferred for the disinfestation of stored commodities, with phosphine (PH_3_) being by far the most widely used gas for the protection of stored grain. The low cost, ease of application, ability to readily penetrate the grain bulk and lack of chemical residues are all characteristics of phosphine that make it an ideal fumigant. Furthermore, it has no effect on grain viability [5].

Alternative fumigants are available, but their use is restricted because they cause environmental damage, leave residues on grain, or have limited efficacy. For example, methyl bromide depletes the stratospheric ozone layer [6], sulfuryl fluoride leaves residues on grain, has limited efficacy against insect eggs [7] and is a potent greenhouse gas [8], whereas ethyl formate cannot penetrate large grain bulks. Due to the limitations of alternative fumigants, phosphine is the only fumigant approved for general use worldwide [9,10], which, when combined with domestic and international market demand for insect-free grain, has resulted in a heavy reliance on phosphine [11].

The overreliance on phosphine has contributed to the selection of phosphine resistance among grain insect pests. Low level phosphine resistance was first observed globally in a 1970s survey conducted by The Food and Agriculture Organization of the United Nations (FAO) for resistance development to insecticides among stored product insect pests. Ten percent of the insects collected worldwide exhibited some degree of phosphine resistance [12]. Currently, reports of high-level phosphine resistance among stored product pests are common around the world [13,14,15,16,17,18,19]. Australia is unique in having a national resistance-monitoring program that has been in place for nearly three decades. This program has documented the origin of highly phosphine-resistant insects from multiple species including the flat grain beetle *Cryptolestes ferrugineus* (Stephens) (Coleoptera: Laemophloeidae), the lesser grain borer *Rhyzopertha dominica* (L.) (Coleoptera: Bostrichidae), a psocid *Liposcelis bostrychophila* (Badonnel) (Psocoptera: Liposcelidae), the red flour beetle *Tribolium castaneum* (Herbst) (Coleoptera: Tenebrionidae) and the rice weevil *Sitophilus oryzae* (L.) (Coleoptera: Curculionidae) [20]. This well-documented development of resistance on a global scale jeopardizes the grain industry’s ability to maintain insect- and residue-free grain, especially with the lack of phosphine alternative fumigants. This precarious situation is shored up by the use of methyl bromide as a quarantine treatment at port facilities and sulfuryl fluoride [21] as an emergency fumigant when phosphine fumigation fails, also carbonyl sulfide has been recently reported as an effective alternative fumigant to control the developmental stages of phosphine-resistant insects [22].

Several treatments have been found to interact with phosphine, either enhancing or reducing its toxicity. Treatments that synergistically enhance phosphine potency may result in more efficient management of stored grain pests. By reducing the dose/concentration required to control pests [23], synergists have the potential to slow insecticide-resistance development in the target pests. Even fumigants that do not interact with phosphine mechanistically may improve fumigation outcomes when applied in combination. This is the case with sulfuryl fluoride, which has a mechanism of toxicity that does not intersect (or overlap) with that of phosphine, but which nonetheless exhibits complementary properties to those of phosphine that improve pest control efficacy: sulfuryl fluoride is fast acting and controls phosphine-resistant insects, whereas phosphine is more effective against the egg life stage and is much less expensive [21,24].

This review highlights the mode of action and mechanism of resistance of phosphine. Moreover, it discusses phosphine interaction with other treatments that usually coexist with phosphine during the grain storage process.

## 2. Phosphine Mechanisms of Action

**Figure 1:**
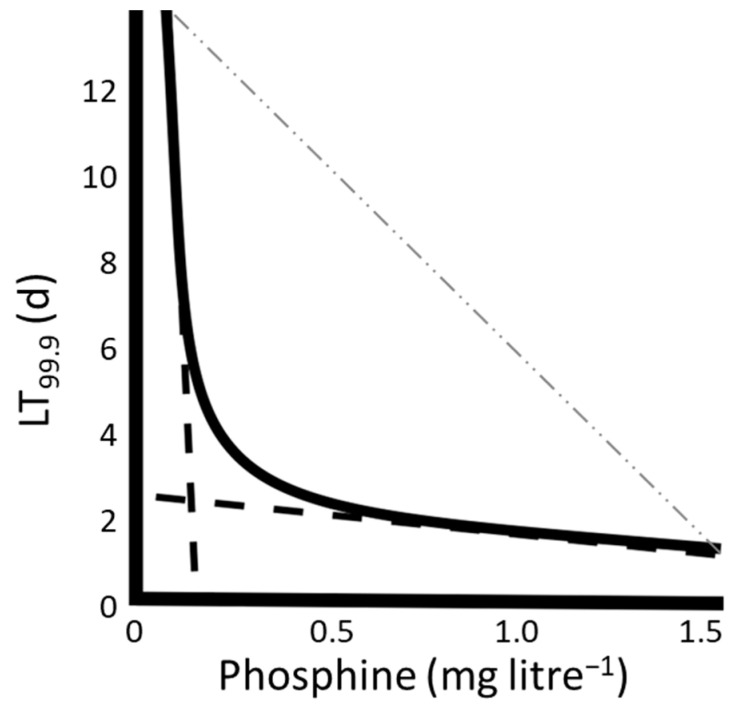
A stylized representation of a non-linear response to phosphine. The days required to achieve 99.0% mortality are plotted on the *y*-axis, whereas the concentration of phosphine to which the insects were exposed is plotted on the *x*-axis. For context, a typical linear dose response curve as observed for typical pesticides is shown as a faint dashed line. The bold dashed lines represent fast and slow modes of action of phosphine that, when combined, result in the observed non-linear response [26].

Nath et al. [25] summarized three proposed mechanisms to explain phosphine toxicity: oxidative stress, metabolic crisis and neurotoxicity. It ought to be noted that the mechanisms proposed are not mutually exclusive, a possibility that is supported by the observation that at very high concentrations, phosphine is a fast-acting toxin, but it is a very slow-acting toxin at low concentrations (Figure 1) [26]. The two modes of action give rise to a non-linear relationship between phosphine concentration and the duration of exposure required to achieve mortality. This situation is in contrast to most pesticides, for which effectiveness is represented by an inverse, linear relationship between concentration and exposure time.

### 2.1. Oxidative Stress

The first proposed mode of action is related to phosphine’s ability to trigger the generation of reactive oxygen species (ROS) in aerobically respiring organisms. These highly reactive oxygen molecules damage biological macromolecules, which if unchecked can lead to cell death. This is a slow form of toxicity that is proposed to be the primary cause of death in pest insects during grain fumigation that can last from days to weeks [27].

ROS are primarily produced as a side reaction of enzymes of energy metabolism involved in electron transfer including the oxidative phosphorylation reactions of energy metabolism. As a result, a high rate of aerobic respiration is associated with increased ROS generation, a situation that is exacerbated by exposure to phosphine [25,28]. The relationship between aerobic respiration and phosphine toxicity is illuminated by the effect of mitochondrial uncouplers. Exposing animals to mitochondrial uncouplers induces an artificially high rate of aerobic respiration that results in an increase in the rate of electron flow through the mitochondrial electron transport chain (ETC). When Valmas et al. [29] co-exposed both wild-type and phosphine-resistant mutants of *Caenorhabditis elegans* to a non-lethal dose of mitochondrial uncoupler plus a non-lethal concentration of phosphine, the combination caused complete mortality of both phosphine susceptible and resistant strains. These findings support a correlation between phosphine toxicity and aerobic respiration rates within the mitochondria [29], which is likely due to an increase in the generation of ROS. The role of ROS as a mediator of phosphine toxicity is supported by the observation that exposure of wild-type *C. elegans* to a non-lethal concentration of phosphine (70 ppm) doubled the toxicity of diethyl maleate when the nematodes were cultured on agar plates containing it before the administration of phosphine. Diethyl maleate is a compound that depletes the major cellular antioxidant, glutathione [30]. Diethyl maleate was subsequently found to enhance the toxicity of phosphine, with a decrease in *C. elegans* fecundity at sublethal exposure to phosphine and an increase in mortality at higher doses [31].

Despite the correlation between the toxicity of phosphine and aerobic respiration, phosphine inhibition of mitochondrial respiration in vitro did not differ between mitochondria isolated from resistant versus susceptible insects [32]. In a different study, the author observed that in vivo exposure to phosphine for a sublethal time resulted in reduced oxygen consumption, but only in susceptible animals [33], which runs counter to the discovery that the respiration rate is positively associated with phosphine toxicity. It is possible that the mechanism of action is to enhance the rate of ROS generation despite inhibition of the respiratory rate if the inhibition occurs in vitro and contributes to phosphine toxicity.

An early observation was that phosphine could disrupt mitochondrial function by inhibiting cytochrome *c* oxidase [34,35], i.e., complex IV of the mitochondrial electron transport chain, a critical component of oxidative phosphorylation and the site of oxygen consumption during aerobic respiration. As a mechanism of toxicity, the interference with aerobic respiration appears to contradict the observation that active respiration is positively correlated with phosphine toxicity. It is possible, however, for a toxic mechanism such as ROS generation to result from either an increased rate of metabolism or dysfunction of a redox enzyme’s activity [36,37,38].

In a histopathological study involving cytochrome *c* oxidase, orally exposing rats to phosphine decreased the activity of mitochondrial complexes I, II, and IV in liver tissue, whereas phosphine intoxication reduced the level of all cytochromes in the treated animals’ livers and brains. The treated rats’ histological changes revealed mitochondrial injury in the heart, liver, and brain tissues, causing a reduction in energy output and elevated oxidative stress. Acute phosphine exposure in rats caused significant suppression of catalase activity, resulting in an increase in lipid peroxidation [39]. This indicates that phosphine interferes with cellular respiration by targeting the mitochondria. Duenas et al. reported an anti-ischemic metabolic agent Trimetazidine that can reduce the toxic effect of phosphine by preserving oxidative metabolism by improving glucose utilization by inhibiting fatty acid metabolism to counteract these insults in phosphine-poised patients [40].

The discovery of the *rph2* resistance factor in 2012 provides an alternative interpretation of the role of aerobic respiration in phosphine toxicity. The *rph2* gene encodes the enzyme dihydrolipoamide dehydrogenase (DLD). This enzyme contributes to five enzyme complexes [41], including pyruvate dehydrogenase, which serves as the metabolic switch between anaerobic and aerobic respiration. DLD and/or the enzyme complexes to which it contributes are potential sources of cellular ROS, particularly the 2-oxoglutarate dehydrogenase complex, which is an integral component of aerobic respiration [42,43]. The product of the DLD-containing enzyme complexes is nicotinamide adenine dinucleotide (NADH), which feeds high-energy electrons to the mitochondrial electron transport chain: The chain terminates in complex IV, which was previously stated to be a likely active site of phosphine toxicity [34]. The actual site of action of phosphine requires resolution.

### 2.2. Metabolic Crisis

Another proposed mode of action for phosphine is the suppression of energy metabolism, with phosphine toxicity resulting in a “metabolic crisis” and death [25]. Research conducted on rats found a phosphine-mediated reduction in aerobic respiration caused an energy crisis due to the difficulty of meeting energy needs through anaerobic respiration [35]. When rats were given phosphine, glucose was synthesized in the liver, indicating that the rate of glucose catabolism in brain tissue was increased. A dramatic decrease in the levels of plasma glucose despite increased synthesis of glucose in the liver also supports the emergence of a metabolic crisis [44,45]. It is worth noting that even sublethal doses of phosphine can trigger rapid and dramatic metabolic suppression in *C. elegans*, to 20% of normal within 1 h [38]. In support of this claim, resistant strains of *T. castaneum* and *R. dominica* were found to have an elevated amount of lipids compared to their susceptible counterparts. The lipids were argued to play a major role in resisting the toxicity of phosphine by providing a reliable energy source for insects to survive phosphine induced-stress and providing a protective environment to the mitochondria [46].

Moreover, the recent identification of phosphine resistance variants clustered around the active site of the DLD enzyme, indicates that phosphine may directly target this enzyme and affect the energy metabolism [41]. The enzymatic product of DLD is NADH. NADH feeds electrons into the mitochondrial electron transport chain, which is ultimately responsible for the generation of Adenosine triphosphate (ATP). Thus, the DLD function is integral to the energy balance of the cell. The role of DLD in energy generation provides a possible mechanism to explain the phosphine-mediated inhibition of aerobic respiration.

### 2.3. Neurotoxicity

A neurotoxin is a third potential mechanism of phosphine toxicity. There is limited evidence that phosphine inhibits acetylcholine esterase activity (AChE). Since esterase activity is required to inhibit acetylcholine signaling, inhibiting the esterase results in elevated levels of synaptic acetylcholine and possibly excitotoxicity [47,48]. In a single, very limited study, Mittra et al. [49] provided direct evidence for cholinergic excitotoxicity in their finding that phosphine toxicity in rats was decreased by co-treatment with pralidoxime and atropine, two compounds that antagonize cholinergic excitotoxicity. The behavioral effects of exposure to phosphine are very rapid [47,48,49,50], indicating that, similar to metabolic disruption, neural disruption can also occur rapidly.

## 3. Phosphine Resistance

In 2002, Collins et al. characterized two strains of *R. dominica*, one weakly resistant and the other strongly resistant to phosphine according to the discriminating doses of the international standard FAO test [12,51]. They showed that strong resistance to phosphine is the product of more than one incompletely recessive gene. Subsequently, two genes were identified in *R. dominica* by Schlipalius et al. [52], which together were responsible for strong resistance. One gene, *rph1*, provides up to 50-fold phosphine resistance, while the other, *rph2*, provides resistance of up to 12-fold. The two loci act synergistically when both are homozygous resistant, resulting in >250-fold phosphine resistance when compared with completely susceptible insects. The same two genes are the primary contributors to high-level resistance in *R. dominica*, *T. castaneum*, *S. oryzae* and *C. ferrugineus* [15,53,54,55,56]. Similarly, in the model organism *C. elegans*, a mutant selected for phosphine resistance was found to carry a missense mutation in the *dld-1* gene (the orthologue of *rph2*). This phosphine-resistant *C. elegans* strain is resistant to phosphine at nine times the basal tolerance of the fully susceptible wild-type strain [41,57].

Gene expression analysis in *C. elegans* indicated that exposure to phosphine triggered a defense response against iron toxicity. Biochemical analysis confirmed that phosphine exposure in *C. elegans* caused an increase in the levels of free iron, contributing to lipid peroxidation and death. Suppression of the iron-sequestering ferritin-2 gene in *C. elegans* increased phosphine sensitivity [58]. The contribution of iron to phosphine toxicity in insects has not been established.

The relationship between phosphine and oxidative phosphorylation was investigated by individually epigenetically suppressing twenty-one mitochondrial ETC genes in *C. elegans*. The inhibition of several of these genes resulted in suppression of the respiration rate and elevation of phosphine resistance in susceptible animals by up to 10-fold compared to the controls. Chronic suppression of aerobic respiration epigenetically or by mutation likely pre-adapts cells to a low energy state, preventing a metabolic crash and energy crisis upon exposure to phosphine. The decreased rate of aerobic respiration could also suppress the generation of ROS [38].

It was previously proposed that the activity of an efflux pump might protect resistant insects from phosphine exposure [59]. Evidence for this was provided by the observation that phosphine-resistant insects take up less phosphine compared to their susceptible counterparts [60]. However, it was subsequently found that the increase in the uptake of phosphine in susceptible insects was correlated with an increase in phosphine oxidation within the cells [61,62]. Oxidation results in increased hydrophilicity, preventing diffusion of the molecule back across the membrane, and leading to an accumulation of labeled phosphorus in the tissues of the susceptible insects. Thus, there is a correlation between phosphine oxidation and susceptibility to its toxicity. While the nature of this relationship is not understood, it provides an alternative explanation to the idea that resistance is due to the activity of an efflux pump in resistant insects. Interestingly, a phosphine-activated efflux pump has been identified, but it is an arsenate efflux pump with no known activity against phosphine [28].

An earlier hypothesis proposed that a narcosis effect observed at higher doses was a phosphine-protective mechanism in resistant insects [5,37,63,64]. Later, when Winks and Waterford [65] discovered that the concentration of phosphine that causes a narcotic effect in *T. castaneum* was ten times higher in resistant animals than in susceptible ones, that claim was dismissed as a cause of phosphine resistance. As a result, narcosis is not a phosphine resistance mechanism.

## 4. Phosphine Interaction with Other Treatments

One approach to understanding the precise mode of action of phosphine is to study the interaction between phosphine and other treatments. For example, oxygen concentration during phosphine fumigation was found to be positively correlated with phosphine toxicity, though the relationship between oxygen and toxicity is not unique to phosphine [66]. When two species of insects were exposed to oxygen during fumigation, the mortality caused by seven fumigants, including phosphine, increased. The oxygen-enhanced toxicity of phosphine, on the other hand, was significantly greater than that of the six other fumigants (hydrogen cyanide, acrylonitrile, methyl bromide, ethylene dibromide, ethylene oxide, and chloropicrin) [67]. When applied up to thirty hours after phosphine fumigation, a high oxygen atmosphere was able to enhance phosphine toxicity [68].

Phosphine toxicity is also affected by a lack of oxygen, with phosphine losing its toxicity under anoxic conditions [36,69]. In fact, under anoxia, the wheat weevil (*S. granarius*) was almost completely protected against phosphine, with more than 22 mg L^−1^ required to achieve the median lethal concentration (LC_50_) compared to 1 mg L^−1^ in the presence of oxygen in normal air [70]. Three other species of stored product pests, *T. castaneum*, *T. confusum* and *R. dominica*, were able to tolerate 10 mg L^−1^ of phosphine for 12 h under anoxia (~98% survival). On the other hand, 2 mg L^−1^ of phosphine was lethal, causing 100% mortality when the oxygen concentration was increased to ≥2% subsequent to phosphine fumigation [69].

The relationship between oxygen and phosphine toxicity is also seen in the model organism *C. elegans* in which the non-lethal concentration of 0.1 mg L^−1^ phosphine at 25 °C for 24 h caused 100% mortality of the wild-type strain when combined with 80% oxygen [28,57]. Additionally, in postharvest control of horticultural insect pests, fumigation with hyperoxia dramatically decreased the exposure time and phosphine concentration required to achieve complete pest control [71,72].

Arsine gas is rapidly oxidized to arsenite, which can interact directly with the lipoic acid cofactor of the four enzyme complexes that contain the phosphine resistance factor DLD [73,74,75]. Arsine susceptibility and phosphine resistance have been found to be positively correlated [41,76]. For example, a phosphine-resistant strain of *R. dominica* was 50% more susceptible to arsine than a phosphine susceptible one [76]. Phosphine-resistant *C. elegans* were also significantly more susceptible to both arsine and arsenite than wild-type animals [41]. Similarly, fumigation of phosphine-resistant *C. elegans* with the sublethal concentration of 70 ppm phosphine increased the mortality caused by 4mM arsenite from 50% to 89%. Interestingly, a phosphine-susceptible strain of *C. elegans* was actually protected from arsenite toxicity due to phosphine activation of an arsenite efflux pump [28].

Synergistic enhancement of phosphine toxicity is not limited to chemicals, as increased temperature also improves the efficacy of phosphine fumigation. In poikilothermic animals such as insects, the rate of respiration is correlated with temperature [77,78], which means that elevated temperature increases the toxicity of phosphine, which is dependent on aerobic respiration. The reverse is true at low temperatures [79]. Another study found that increasing the temperature reduced the time to population extinction (TPE) for the phosphine-resistant psocid, *L. bostrychophila*. The 11 days TPE with 1 mg L^−1^ of phosphine at 15 °C has been reduced to only two days when the fumigation temperature increased to 35 °C [80].

Phosphine fumigation is typically carried out in the presence of 2–3% carbon dioxide (CO_2_), which decreases the chance of spontaneous combustion. A much higher CO_2_ concentration of 29% also synergistically enhances phosphine toxicity [81]. The conventional phosphine concentration range for achieving complete mortality in mills was decreased from 850–1500 ppm to 65–165 ppm when a combination of high temperature (32–37 °C) and 4–6% of CO_2_ was applied with phosphine fumigation [82].

In the standard atmosphere, high temperature (37–40 °C) is stressful to many insects [83], so combining it with other physical stressors will amplify the damaging effects. Mbata and Phillips [84] increased the toxicity of low pressure to stored product insects by conducting their experiment at high temperatures. The estimated lethal time LT90 for *R. dominica* larvae in low pressure was 64 h at 25 °C, while the LT90 decreased to 5 h at a high temperature of 40 °C. In their discussion, they implied that high temperatures increase respiration and metabolic rates, resulting in rapid mortality among exposed insects [84]. Remarkably, exposure to high temperatures before fumigation with phosphine decreases phosphine toxicity. The stress from high temperature exposure induces resistance against phosphine that can exceed 3-fold [85].

Integrated pest management (IPM) contributes significantly to managing phosphine resistance. One of the approaches is ionizing radiation, including the use of gamma radiation. This approach has gained an excellent reputation as a residue-free treatment in stored-product pest management [86,87,88,89,90,91,92,93,94]. While it has not been extensively used for grain protection, treating wheat infested with immature stages of *R. dominica* with 250 Gy of gamma-ray decreased the rate of adult emergence by 54% compared with the controls [93], but sterility and ultimate mortality were not assessed. Exposing *S. granaries* eggs to 30–500 Gy caused development inhibition, which prevented adult emergence [86]. The mortality of *T. confusum* adults reached 99% at 30 days from the time of exposure to 200 Gy of gamma radiation [90].

In dates, the disinfestation of *Oryzaphilus surinamensis* was efficiently achieved with gamma irradiation. Seven hundred Gy of gamma radiation was an optimal dose for controlling all developmental stages of the insect. Remarkably, only 85 Gy was enough to sterilize this pest and inhibit reproduction [94]. Exposing eggs of *Ephestia kuehniella* to 400 Gy reduced the hatchability to 27%, and no adult emerged from the hatched eggs [89]. The inhibitory effect of gamma radiation was observable in *Plodia interpunctella* immature stages, which failed to develop when irradiated with 500 Gy [88].

In addition to routine pest control, many countries utilize gamma irradiation as a quarantine treatment to disinfest commodities for import and export [91,95,96]. In these countries, gamma radiation usually co-exists with phosphine in stored-product pest management. This co-existence generated interest in a potential interaction between the two treatments, but the interaction between phosphine and gamma radiation was not observed in either susceptible or resistant strains of *T. castaneum* [97]. The strain used in this study was only weakly resistant to phosphine, and the study was carried out at a time when the resistance at the *rph1* locus predominated. However, cross-resistance to gamma radiation of a phosphine-resistant strain of *R. dominica* was observed [98].

On an experimental scale, ultraviolet radiation has been investigated as an approach for stored product pest control and as a hygiene treatment [99,100,101,102]. UV radiation can stop the development process of the khapra beetle *Trogoderma. granarium* at various stages. One hundred percent mortality was achieved after irradiating the eggs with 56.52 J cm^−2^ of UV light. The radiation caused damage to the eggs’ chorions, resulting in a leakage of the inner contents. Other juvenile stages of this pest were sensitive to UV, and the same dose produces 98.3% and 91.7% mortality in larvae and pupae, respectively [102]. However, the interaction between UV and phosphine has not been looked at, probably due to the practical limitations of using UV in grain protection. UV light cannot penetrate the grain, which shields grain pests from the lethal effects of UV exposure.

Besides direct DNA damage, radiation can damage a wide array of cellular molecules through the generation of ROS. Antioxidant enzymes such as catalase and superoxide dismutase play an essential role in cellular defenses against radiation-induced damage [103] and significantly protect cells in the exposed organism from the damaging effect of UV radiation [104]. The oxidative stress caused by ionizing radiation can also be reduced by the antioxidant resveratrol, which decreases cellular damage [105]. The relationship between oxidative stress and radiation exposure is observed as a negative correlation between the levels of ROS in cells and their radioresistance [106].

**Figure 2:**
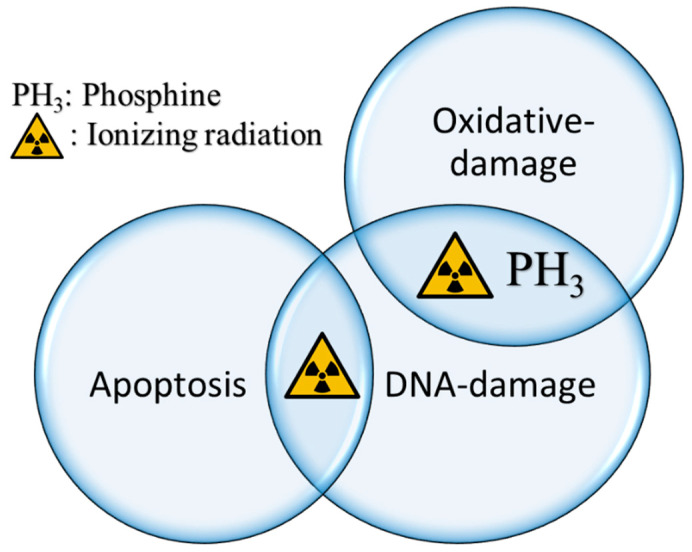
Ionizing radiation results in physical damage to DNA as well as high levels of oxidative stress. Phosphine also causes significant oxidative stress and has been shown to damage DNA [106]. A *C. elegans* strain that is resistant to radiation induced apoptosis does not show cross-resistance to phosphine. This indicates that the superficial similarity between the modes of action of phosphine and ionizing radiation is not reflected in a shared antiapoptotic resistance mechanism.

Since phosphine has been identified as a redox-active toxin that generates significant oxidative stress [5,25], there is a possible overlap between the biological pathways of the toxic action of the treatments (Figure 2). This event was reported by [107], where the phosphine-resistant strain of *C. elegans* showed cross-resistance to UV and gamma radiation. Interestingly, mutations that cause a deficiency in DNA repair, which causes hypersensitivity to radiation also exhibit hypersensitivity to phosphine [107]. In another study, exposing the nematodes to a mild dose of UV or gamma radiation prior to phosphine fumigation elevated the animals’ tolerance against the fumigant [85].

## 5. Summary

Continuous use of phosphine, due to the lack of suitable alternatives for fumigating stored grain, has resulted in highly resistant insect pests. Therefore, understanding phosphine’s mode of action and the resistance mechanisms is essential to maintain its effectiveness for the protection of stored grain. The most strongly supported mode of action of phosphine is the generation of reactive oxygen species by respiratory enzymes, which results in oxidative damage to cellular systems. Another possibility that is not mutually exclusive is the suppression of energy metabolism, resulting in an energy crisis in the exposed organism, particularly when the energy deficit is coupled with the energy demands imposed by cellular repair systems. The inhibition of cytochrome *c* oxidase by phosphine may also contribute to phosphine toxicity, but the evidence is equivocal. In addition, phosphine exposure triggers hyperactivity followed by narcotic effects consistent with neurotoxicity. Indeed, exposure to phosphine can lead to the inhibition of AChE causing an increase in acetylcholine neurotransmission and possible excitotoxicity.

Phosphine resistance in pest insects of stored grain is governed by two genes, *rph1* and *rph2*. Individually, each of these two genes provides weak resistance to phosphine. When combined, however, resistance increases synergistically. In their normal forms, the *rph1* gene increases the sensitivity of cellular membranes to ROS and the *rph2* gene contributes to ROS generation.

The resistance variants of these genes likely suppress ROS generation and sensitivity, explaining the interaction between the two resistance genes. The *rph2* gene in particular is a major contributor to aerobic energy metabolism, which, when disrupted by phosphine, could contribute to a crisis of energy metabolism. The pyruvate dehydrogenase enzyme complex that employs the DLD enzyme encoded by the *rph2* gene as one of its subunits generates acetyl CoA, a precursor of acetylcholine. Thus, the cellular oxidative stress, energy crisis and excitotoxicity mechanisms of phosphine toxicity may all be mediated through direct interaction with the DLD enzyme encoded by the *rph2* resistance factor. Recent insights into synergists, induced tolerance and resistance factors are also consistent with an interaction between phosphine and the DLD enzyme. Arsenite, for example, is able to interact in a direct way with the lipoic acid cofactor of the four enzyme complexes that contain the phosphine resistance factor, DLD. Arsine susceptibility and phosphine resistance have been found to be positively correlated. Oxygen, moreover, enhances phosphine toxicity if it is introduced during, before, or after phosphine fumigation. Phosphine toxicity is also affected by a lack of oxygen, with phosphine losing its toxicity under anoxic conditions. High temperature elevates the respiration rate, increasing the toxicity of phosphine, which is dependent on aerobic respiration. Phosphine fumigation is typically carried out in the presence of 2–3% CO_2_, which decreases the chance of spontaneous combustion. A much higher CO_2_ concentration of 29% also synergistically enhances phosphine toxicity. High temperature exposure before fumigation induces resistance against phosphine that can exceed 3-fold. Phosphine and ionizing radiation can co-exist in the grain industry process. The two toxins overlap in their toxic pathway by generating oxidative stress leading to cross-susceptibility between the two treatments. However, pretreatment with radiation can enhance phosphine resistance.

## 6. Future Directions

More biochemical studies are required to determine the precise mode of action of phosphine. The mechanisms of resistance also need to be precisely ascertained to enhance the management of resistance in economic pests. In resistance management, investigating and introducing phosphine alternatives to be rotated with phosphine can significantly integrate in reducing the development of resistance. From a general health perspective, a phosphine antidote needs to be developed to reduce or eliminate the toxic effect of phosphine, but this cannot be achieved without determining the precise mode of action.

## Data Availability

No new data were created or analyzed in this study. Data sharing is not applicable to this article.
